# Simulating employment and fiscal effects of public investment in high-quality universal childcare in the UK

**DOI:** 10.1186/s40723-022-00096-y

**Published:** 2022-02-14

**Authors:** Jerome De Henau

**Affiliations:** Faculty of Arts and Social Sciences, The Open University, Walton Hall, Milton Keynes, MK7 6AA UK

**Keywords:** Free universal childcare, Social infrastructure, Improved quality, Macro-level employment effects, Fiscal revenue, Gender equality

## Abstract

This paper simulates the likely fiscal and employment effects of a vast public annual investment programme of free universal high-quality early childhood education and care (ECEC) services in the UK. It examines the extent to which it would pay for itself fiscally for different scenarios of pay increases. Investing in high-quality universal ECEC benefits all children by improving their life chances, especially for those living in lower income families. It also generates larger employment effects than other more typical investment policies such as construction projects and fosters gender equality in employment: not only it provides many high-quality jobs for women, it also allows many mothers to improve their lifetime earnings prospects by freeing up their childcare constraints. This in turn has beneficial fiscal revenue effects for the government. Estimations of annual public expenditure for a system of highly qualified and well-paid childcare staff with low child-to-staff ratios are performed, with universal coverage for all pre-school children aged 6 months to 4.5 years. Labour demand and matching supply effects are also simulated using input–output methods, for different take-up rates of the programme. A microsimulation tool is used to calculate increases in household income and tax liabilities and decreases in social security benefits spending. This results in a net annual funding requirement of between 28 and 39% of the gross investment. Two funding methods are then explored: raising taxation in a progressive way and recouping the cost over time from persistent mothers’ increased earnings. The former would entail a net additional contribution by the richest 20% of households of at most 0.4% of their income; the latter would require 21 to 31 years to offset the programme on average, which is within a typical working life-course following a first child’s birth, of 35 years.

## Introduction

This paper investigates the potential employment and fiscal effects of investing in free universal early childhood education and care provision (hereafter ECEC or childcare) as one of the bedrocks of the social infrastructure that is currently deficient in the UK and in many other OECD countries (OECD, 2017). Social infrastructure—the systems of care, health and education services—is essential to achieve a sustainable economy and require significant public funding to avoid being underprovided (De Henau & Himmelweit, 2021; Elson, 2017; Ilkkaraçan, 2017). In the aftermath of the 2008 financial crash, many governments across the developed world, reacted to the crisis in public finances by cutting many public services that build the social infrastructure without effective replacement of equivalent quality in the private or voluntary sector (Bargawi et al., 2017). Moreover, many of the childcare services in the UK already lacked the level of accessibility, affordability and quality that the economy required, even prior to the crisis (Cory, 2017). The COVID-19 pandemic has shown both how vital the sector was for children and their mothers after taking the brunt of ‘home’ schooling, and how government did indeed have sufficient resources that could be deployed at short notice to inject cash in the economy. This has triggered renewed public support for government investment in essential public services of which childcare was seen as core alongside health, education and adult social care (Heintz et al., 2021).

Yet, despite the easing in austerity stances and a renewed focus on public investment in the UK and elsewhere in Europe and the US in the wake of the COVID-19 pandemic, priority for government remains to seek traditional routes of physical infrastructure investment. Despite women having borne the brunt of the effects of lockdowns and pressure on public services, current solutions advanced leave them largely out of the picture (De Henau & Himmelweit, 2021). Governments remain cautious when it comes to funding public services that are seen as ‘consumption’ expenditure. The push for social investment in ECEC promoted by the European Commission in the 1990s, such as investing in the future productivity of children, has always been tempered by concerns for fiscal orthodoxy and a rhetoric of private choice for families, especially in more liberal welfare regimes such as the UK (De Henau & Himmelweit, 2013). As a result, ECEC policy in the UK remains inadequate. Despite investment by the government in different schemes over the last twenty years, a hybrid market-based system of free direct provision for some families, cash subsidies for low-income families, and tax relief for others, is not only complex to navigate but remains largely underfunded. This has produced an odd result of childcare costs being among the highest in Europe for couple families on middle incomes (OECD, 2020) with low-qualified staff on low pay, as private providers try to push down costs to remain on the market (Eurofound, 2015).

As calls for a total overhaul of the system are gathering pace (despite not being new) this paper examines the following idea: investing sufficient funds for providing all children with adequate childcare of high quality, free for their parents at the point of use and regardless of their circumstances, can be done without draining public finances.

The next section reviews the literature to make the case in favour of universal childcare of high quality and why it would address the issues specific to the various systems of funding in the UK. It is then followed by a section outlining the simulation strategy of how investing in ECEC services would increase employment and fiscal revenue. I then explain the method used to calculate the annual public expenditure required to provide universal childcare according to different mixes of staff pay and qualification levels. From this investment, I can estimate the aggregate employment effects of labour demand and supply. On the labour demand-side, standard input–output methods are used to simulate indirect employment effects (from industries supplying the childcare sector) as well as induced employment effects (stemming from increased household consumption in the wider economy). I go on to discuss the particularities of such investment in relieving some of the constraints the carers face to finding decent full-time employment should they wish to. By potentially increasing labour supply as well as labour demand, such investment, unlike say an investment of equivalent magnitude in physical infrastructure, may be more efficient in achieving full employment, especially in a context of low unemployment but high underemployment (especially for women). The fiscal impact of increased employment and improved working conditions, especially among mothers of young children, is then discussed and estimated in both a static and a dynamic framework, using a tax-benefit microsimulation tool. The last section discusses the policy implications of such results and concludes.

## Making the case for free universal high-quality ECEC provision

A large pool of international research findings have shown that access to formal childcare—provided it is of high quality and for a significant number of hours during the week—is crucial to improving children’s outcomes and life chances, even for very young toddlers and infants, especially those from more disadvantaged backgrounds (van Huizen & Plantenga, 2018; Ünver et al., 2018; Petitclerc et al., 2017; Huston et al., 2015; Sibley et al., 2015; Dearing et al., 2015; Havnes & Mogstad, 2011, 2014; Lloyd & Potter, 2014; Bauchmüller et al., 2014; Babchishin et al., 2013; Li et al., 2013; Stephen et al., 2011; Penn, 2009).

Another strand of childcare literature examined the benefits of such universal policies for maternal employment, concluding that mothers’ labour supply (but not fathers’) is greatly improved by access to high-quality, universal, low-fee childcare of significant length, especially for women in couple. Empirical analyses of policy reforms around the turn of the 21st Century have shown this for Norway (Andresen & Havnes, 2018), England (Brewer et al., 2020), the Canadian province of Quebec (Lefebvre et al., 2009; Fortin, 2018) and across other economies (Huston et al., 2015; Penn, 2009; van Huizen & Plantenga, 2018).

ECEC provision is also seen as contributing to increased employment overall and reduced gender inequalities in income over the life course (De Henau & Himmelweit, 2013; Garcia et al., 2016; Kim et al., 2019; Kleven et al., 2019a).

Yet despite successive government interventions in the sector, ECEC provision in the UK is still largely inaccessible and unaffordable to many parents, and of uneven quality (Harding & Cottell, 2018; Lloyd, 2018). The cost to parents is very high in the UK compared to its European neighbours and cost rises have been outstripping general inflation over the last fifteen years (Butler & Rutter, 2016; Harding & Cottell, 2018; OECD, 2020). Reports analysing UK ECEC provision also point to the lack of places for young children, even among private providers, while state support necessary to make a childcare system viable remains too low or inadequate (Cory, 2017; Harding & Cottell, 2018).

The system consists of a complex mix of direct subsidies to providers (which vary between the four nations—England, Scotland, Wales and Northern Ireland), tax breaks for families and cash support to low-income families. In England, public subsidies to providers to offer free childcare for all 3–4 year-olds (and about 40% of 2 year-olds, from disadvantaged families) only cover 15 h a week during school term (38 weeks of the year). Moreover, the payment to providers per hour of childcare is deemed to be below their supply cost. This has led them to recoup the shortfall by raising fees for hours purchased by parents, increasing the already high costs of UK childcare yet further. Since 2017 the increase to 30 h of free childcare for working parents risk compounding this problem as funding has remained inadequate (House of Commons, 2018). In addition, a complex system of means-tested cash transfers (tax credits) to low-income families with children, including subsidies to pay for childcare expenses, leads to heavy costs being borne by second earners if they work more than short part-time weeks (De Henau, 2017). Despite these forms of support targeted at disadvantaged families, the UK system is characterised by higher levels of inequality in use compared to more universal systems (Petitclerc et al., 2017; Sibley et al., 2015; Van Lancker, 2013). Analysis in other countries has also shown that supply-side investment (direct subsidies) was more cost-effective than demand-side cash support to families in increasing children’s enrolment in ECEC, given capacity limitations (Aran et al., 2018). But this is true only if these subsidies are covering all the costs: the experience of Quebec showed that cutting corners to address increased waiting lists after the introduction of universal low-fee childcare entitlements by providing underfunded subsidies to private providers did not work, as it pushed down quality with damaging outcomes for children (Fortin, 2018).

This paper looks at the costing and funding possibilities of such investment for children aged between 6 months and 4.5 years of age (the average age at which they enter primary school) in the UK. It contributes to the literature on costing and funding universal childcare systems. A study by the New Economics Foundation in 2014 looked at various scenarios (higher pay and qualification) of free ECEC provision for all children aged 6 months to up to 3 years in England (Mohun Himmelweit et al., 2014). Ben-Galim (2011) investigated universal provision and fiscal revenue stemming from increased maternal labour supply but did not consider improved quality standards. Butler and Rutter (2016) proposed a more modest 15-h a week free universal childcare system for all 2–4 year-olds, extended to 30 h for those with working parents and subsidised means-tested fees for additional hours beyond 30 or for younger children (aged 1). They did not examine employment and fiscal effects.

In other countries, Kim et al. (2019) examined the case of extending ECEC provision in Turkey to reach OECD coverage rates but did not examine changes in the quality of the provision. They complemented their direct costing exercise with simulations of employment and fiscal effects and found that about 19% of the investment can be recouped by tax revenue stemming from increased employment. By contrast, Fortin et al. (2013) calculated the fiscal revenue in 2008 from increased maternal employment and economic activity overall following the gradual introduction of low-fee universal childcare entitlement in the Canadian province of Quebec in 1997. They concluded that the net fiscal benefits of the scheme were about 50% higher than the net spending of the programme.

This paper extends the UK studies examining costs in a number of ways. It combines all the parameters of a fully universal full-time high-quality system for all pre-school children, that is increasing free provision as well as pay and qualifications. It also adds essential dimensions to understand the fuller short-term economic effects, based on the findings in other countries. The paper extends the method developed for Turkey, firstly by looking at the wider employment effects, i.e. not just indirect but also induced employment creation in the wider economy and, secondly, by estimating fiscal revenue generated, not just from income and expenditure taxes but also from interactions with the means-tested benefit system, using a reputable tax-benefits microsimulation tool for the UK, the UKMOD tool. It also provides estimations of longitudinal fiscal revenues stemming from increased earnings of mothers over time, following the findings of persistent employment effects of universal childcare policies in Norway and Quebec (Andresen & Havnes, 2018; Lefebvre et al., 2009). Unlike these studies which examined a natural experiment of a policy that had taken place, this paper focuses on appraising potential effects of government childcare policy by modelling various scenarios of take-up and pay rises. This allows us to examine potential trade-offs between quality and ‘affordability’.

## Overview of the simulation strategy

This simulation exercise aims to answer the following question: if the UK government is to overhaul the currently inadequate, expensive and uneven ECEC system and replace it by direct public investment in a free-at-the-point-of-use, high-quality, universal full-time provision, how much would it cost, how can it be paid for and by whom?

The rationale is as follows: not only investing directly in accessible, affordable and high-quality ECEC services increases labour demand (Kim et al., 2019; De Henau et al., 2016), it also makes it more attractive to parents and thus greatly reduce mothers’ labour supply constraints, which mean they can remain in employment after their child’s birth while others can join the labour force (Andresen & Havnes, 2018; Huston et al., 2015; Kleven et al., 2019a; Kornstad & Thoresen, 2007; Lefebvre & Merrigan, 2008; Løkken et al., 2018; Vanleenhove, 2013).^1^

**Fig. 1:**
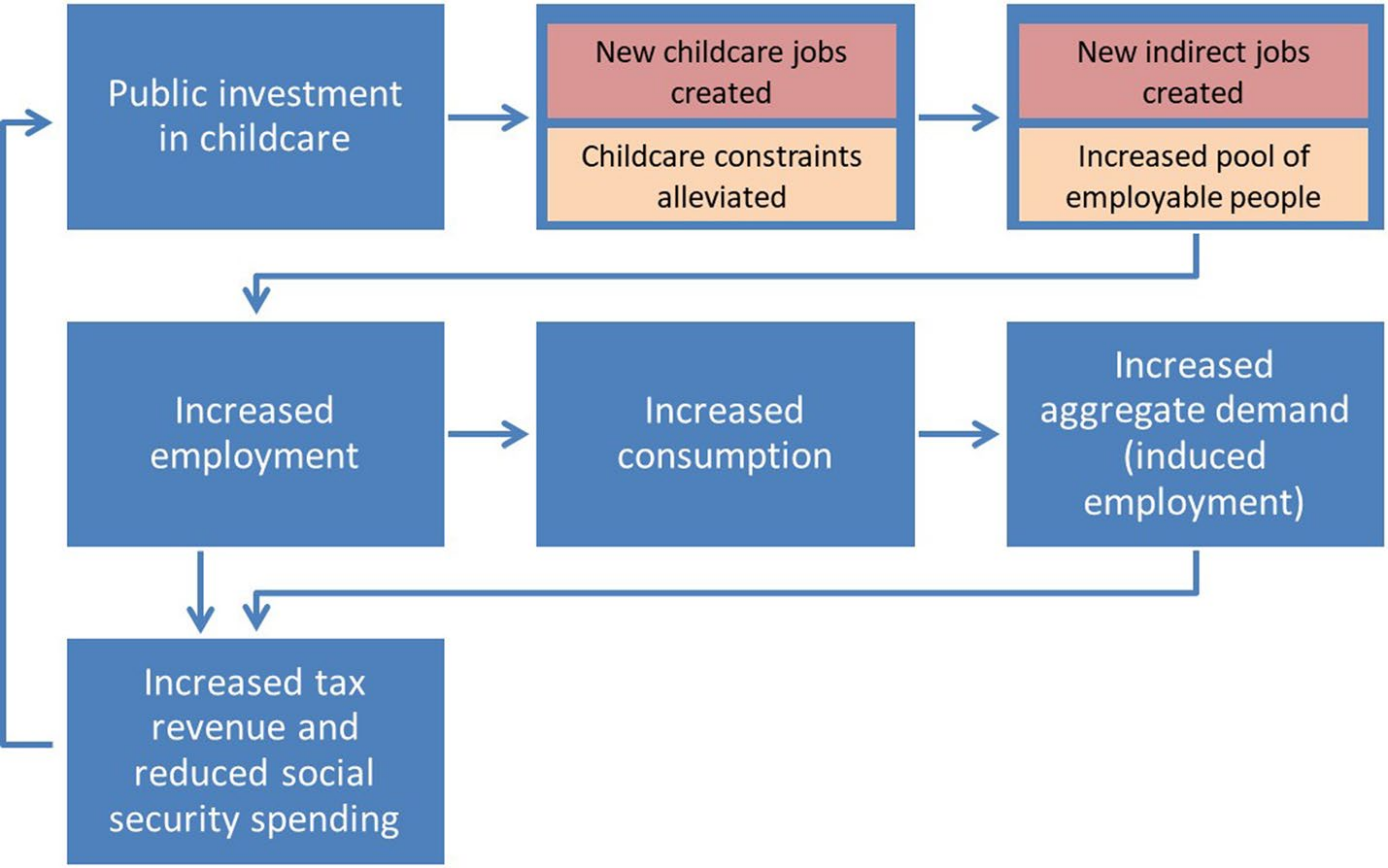
Employment and fiscal effects of investing in universal ECEC services

This increase in employment and retaining of jobs by mothers produces increased earnings overall which leads to higher tax revenue for government and reduces spending on family-related and unemployment means-tested benefits. Simulating various scenarios of costs and the implied potential benefits will help determine the extent to which such effects make the system self-funding over time, that is without the need of raising tax rates. Figure 1 illustrates the conceptual framework of these mechanisms.

Admittedly, the literature also shows that the long-term benefits are not just of maternal employment. Perhaps the greatest benefit with economic implications stems from improvements in children’s outcomes as discussed above, in terms of well-being and educational attainment (reduction in attainment gaps) and thus their employment prospects and reduced public spending on social remediation services. Estimating these longer-term effects are beyond the scope of this paper, though it is worth noting that these benefits will likely result in further net fiscal gains that make the programme even more self-funding (Garcia et al., 2016).

The first stage of the analysis is to estimate the full annual costs of investing in a universal ECEC service and cater for scenarios involving higher qualification and pay for childcare staff compared to the current system. It includes not only running costs such as staff pay and overheads but also provision for training new and existing staff as well as construction costs of new facilities. The parameters for this costing calculation rely on needs identified in the literature of what constitutes good provision, rather than what is currently existing, since this is below standards.

The second stage of the analysis is to simulate employment effects. On the labour market demand-side, employment will not only be directly created in the childcare industry but also in the wider economy, that is in the supply chain of the childcare industry—known as indirect employment—and as a result of increased consumption by the newly employed people—known as induced employment (Scottish Government, 2019). On the supply-side, the people whose employment opportunities (and attitudes to work) are most likely to change with universal ECEC provision include:

- those whose childcare constraints (in time and money) were too high for them to supply their labour (mainly mothers of young children),
- those unemployed or discouraged workers whose qualifications were too low and can now be upskilled by the programme since the investment in childcare includes provision for training.

Following Kim et al. (2019) and Ilkkaraçan and Kim (2019), I assume that in the short-term, labour demand matches labour supply, and all jobs are allocated through reshuffling within the labour market if necessary. This is a plausible assumption for the following reasons:

- The UK labour market is fairly flexible, enabling rapid changes and reshuffles at relatively low transaction costs.
- The model enforces direct government provision, which means the job creation does not rely on the willingness to privately absorb new demand for childcare.
- Provision for training and higher wages also makes the new system more attractive overall than it is currently.
- The model assumes take-up rates below 100% enabling better matching between demand for childcare and employment patterns, with staffing of facilities responding to such demand.

This is not to say that mothers of young children would only fill the new jobs created in childcare. More likely they will retain the wide range of jobs and working patterns they held before having a child while the new jobs created would be filled by changes within the current labour market (e.g. by those who had replaced any job left by women when they became mothers) and a reduction in unemployment.

The third stage of the analysis is to derive the amount of tax revenue and reduced spending on means-tested benefits that stems from these combined employment effects. I estimate likely changes in labour supply of mothers of young children and childless unemployed individuals, based on observable characteristics, and derive new working hours and earnings that match the available job creation on aggregate. I then use a microsimulation tool that re-calculates income tax and social security contributions liabilities and cash benefit entitlements of the whole population, as well as indirect/expenditure taxes derived from increased household income, taking into account the propensity to consume of various income groups.

The fourth stage of the analysis is to compare those costs and revenues to gauge the fiscal sustainability of the programme. One way is to simulate adjustments in tax policy to fund any shortfall, bearing in mind equity and efficiency concerns. This is done using the same microsimulation tool. However, as identified in the literature (Garcia et al., 2016; Kleven et al., 2019a; Lefebvre et al., 2009), persistent labour supply effects for mothers mean that the employment and earnings gains from universal childcare programmes yield fiscal revenue beyond the duration of the programme for the children of the mothers concerned. These longer-term benefits for mothers can be compared to the total costs of the programme for their children (e.g. four years of childcare provision for one child, eight years for two children, etc.). Therefore, rather than increasing tax rates to pay for the annual shortfall or introducing parental fees, I also simply calculate the number of years it would take on average for the programme to pay for itself under the existing tax schedule.

The following sections detail the methods, data and results of each of these stages sequentially, before discussing policy implications and avenues for future research in the concluding section. Figures for costings and taxation relate to the fiscal year 2018–19 (April to March), the latest full fiscal year prior to the major COVID-19 induced disruptions.

## Method for calculating the investment in universal childcare

The model of childcare provision considered here assumes a typical facility that is group-based (crèche, nursery or kindergarten) attended by children of different age groups. This is indeed the dominant form of childcare provision used by parents in the UK and other European countries (Eurydice, 2019). Facilities are age-integrated, as is the case in Norway, Denmark and Sweden, to ease accessibility (Ünver et al., 2018), and develop children’s social skills to smoothen their transition to primary school (Datta-Gupta & Simonsen, 2007).

In such type of facility, the annual amount to invest in ECEC provision for all pre-school children depends on three sets of parameters:

1. Coverage (number of children to be offered a place and opening hours), and take-up rates
2. Staffing requirements, in particular:
  o Ratio of number of children per qualified and other childcare staff, which typically increases with the child’s age
  o Non-contact time of staff (for training and admin work)
  o Level of remuneration and qualification of staff (including cost of initial training and on-costs for pension provision, holiday pay and other social security contributions)
3. Non-childcare staff costs (overhead), including building costs (construction/rent/maintenance). This also includes costs for support activities (cleaning, catering, etc.).

### Coverage and opening hours

This model of universal provision assumes that a place is offered to all children from the age of six months^2^ to the age at which they enter primary school, on average 4.5 years of age in the UK (the year they turn 5). This would significantly extend current provision, especially for children under 3, as only about a third of 0–2 year-olds used formal childcare in 2017, on average for 16 h per week (Eurostat, 2019). Although older children are enrolled at much higher rates, the average number of hours they attend facilities was also low at about 21 h in 2017, compared to over 30 h in the rest of the EU (Eurostat, 2019).

Opening hours of free childcare are assumed to be 40 h per week for children of any age, to account for a full-time working week and commuting time. A full-time working week is assumed to be 35 h, just above the average 34 h per week of women who were employed full-time in 2018 (ONS, 2019a).^3^ The free entitlement is also assumed to be available for 48 weeks per year, allowing for a conservative 4-week holiday period taken between parents. These parameters are more generous than some other proposals (e.g. Butler & Rutter, 2016), or of systems in other countries. For example, many Scandinavian countries offer universal provision but with a low maximum fee (often reduced for lower income families) (Eurydice, 2019).

The calculations in this simulation assume a maximum scenario of fully universal full-time free-at-the-point-of-use provision regardless of employment and income conditions of parents. This is to avoid distorting incentives in both childcare take-up and employment. In essence it extends the model of tax-funded free education to all young children. However, the model assumes less than 100% take-up rates for younger children, based on two scenarios.

The first scenario is a modest but plausible assumption of 50% take-up among children aged 6 months and 1 year, 75% among children aged 2 and 90% among children aged 3 and 4. These reflect current age-specific take-up of non-parental formal and informal childcare as well as take-up rate of free formal care among eligible children (Department for Education, 2018b, 2019), on the grounds that, with improved quality and affordability, parents will substitute (short) informal care for (longer) formal care.^4^ This gives a weighted average take-up rate of 71%.

The second scenario assumes higher take-up rates, at about 67% for children under 2, 90% for those aged 2 and 100% for the 3 and 4 year-olds. This reflects take-up rates of the more generous Nordic countries of Denmark, Iceland and Norway (Eurydice, 2019), which the system being modelled is aspiring to resemble. This gives a weighted take-up rate of 85%.

### Staffing requirements

As childcare provision is a labour-intensive service, childcare staff costs are the largest contributor to total running costs. The number of staff required per facility is determined by the regulatory child/staff ratios for each child’s age group. These statutory ratios differ between countries but are typically lower the younger the child. Most facilities in England currently provide more staff per child than what statutory ratios require, to allow for sickness, breaks and specific children’s additional needs (Department for Education, 2015). The model assumes the more generous ‘current’ child/staff ratios to maximise quality outcomes and minimise disruptions. Table 1 shows the different ratios by age group and the distribution of children by centre according to the assumed take-up rate. Centres cater for an average of 55 children, small enough to retain an attractive ‘home’ environment.

**Table 1:** Child/staff ratios and distribution of children per facility

	Child/staff ratio		No. of children per age	
	Current	Statutory	71% take-up	85% take-up
6 month- and 1 year-olds	2.5	3	15	17
2 year-olds	3.5	4	15	14
3 and 4 year-olds	6	8	25	24

Source: Department for Education (2015, 2018a), ONS (2019b) and author’s calculations

Staff in the new settings are assumed to work 35 h a week following the model’s norm of full-time working hours. This includes contact time with children as well as non-contact time to deal with administrative matters and other business (training, parents, social services). Non-contact time is estimated to be 16% of staff contact time and is used in the model (following Mohun Himmelweit et al., 2014).

Table 2 shows the mix of qualifications found in existing (commercial and voluntary) facilities and their corresponding hourly wage rate (using 2018 data). It shows that the vast majority of staff (57%) was qualified at A-level (Upper secondary school) and very few childcare workers had at least a Bachelor’s degree (13%). By contrast our model of a high-quality universal provision assumes a different mix, whereby only two levels of qualification are considered: supervisory/main ‘teaching’ staff (which on average should account for 45% of childcare workers across age groups in a typical facility according to regulatory ratios) are at a graduate level of qualification (level 6—Bachelor’s degree) whereas all remaining non-supervisory staff are at least at level 3 (A-level—upper secondary school or equivalent with childcare training certificate).

**Table 2:** Distribution of qualification levels and staff gross pay (2018)

	Distribution of staff by qualification level		£ Hourly pay by qualification level	
	Modelled (%)	Current (%)	Teacher	Current
Up to Level 2	0	19	12.77	7.83
Level 3 (A-level)	55	57	15.00	9.20
Level 4 or 5	0	11	17.61	10.8
Level 6 (degree) or above	45	13	19.81	12.3

Source: Department for Education (2018a) and author’s calculations. A-level is roughly equivalent to upper secondary education. ‘Teacher’ pay scale assumes pay scales of primary schools. ‘Current’ pay scale assumes current pay for corresponding qualification levels

Table 2 also shows two different hourly pay scales that are considered in the model. The ‘current’ pay scale shows that staff at lower levels of qualification (level 3) are paid on average £9.20, which is equivalent to the 2018 Real Living Wage (weighted to account for higher wages for London), as calculated by the Living Wage Foundation (2019).^5^ A second pay scale entails a more generous package for each qualification than current wages (second to last column). This is based on the pay scale of primary school teachers, using similar pay levels for equivalent qualifications: as such level 6 childcare workers would be paid £19.81 an hour and level 3 childcare workers £15, equivalent to the corresponding hourly pay of primary school teachers with similar qualifications (accounting for equivalent working hours annually). This is 60% higher than their respective hourly rate in existing commercial facilities. By comparison the median wage rate of all UK employees prevailing in 2018 was £12.77 (ONS, 2019c).

The other staff-related cost elements to take into account are:

- provision for sickness and holiday to replace absent staff, estimated at 10% of contact time in current facilities (assumed throughout all scenarios of the model);
- provision for pension contributions at 14.1% of gross salary (as per level found in the public sector) and employer’s social security contributions (National Insurance);
- provision for training costs (initial and recurring), on average set at 1% of gross pay in current facilities. The model requires a boost to 1.8% of gross pay in the high-qualification option in order to fund initial degree-level training to 45% of staff, based on total costs of university degrees (public subsidy and private fees) annualised over 20 years (Allen et al., 2016).

### Overheads

Overheads are based on non-staff costs in current group-based private and school-based settings, found to be around 27% (Department for Education, 2018b). In the modelled universal setting, overhead costs include provision for repayments of a mortgage for acquiring or constructing the actual buildings, accounting for about 10% of total setting costs, or 40% of overheads. Overhead costs are assumed to be fixed across the different pay and qualification scenarios and set at 25% of total costs of current commercial settings of equivalent capacity/provision, that is a fixed £132,000 per facility per year. This is a plausible assumption given that staff pay and qualification should not influence food, heating and playground space requirements for the children whose number is fixed per centre.

### Overall costing for different scenarios

Using the parameters above, several scenarios for total annual expenditure can be simulated by adding up all costs, which vary by level of staff pay and take-up rates. Table 3 shows the total gross annual investment of providing universal childcare according to these different scenarios. The investment covers 2.3 million children spread over 42,400 facilities in the 71% take-up scenario and 2.8 million children over 50,700 facilities in the 85% take-up scenario, across all four nations of the UK.

**Table 3:** Gross annual investment of universal childcare for different scenarios

	Scenario 1	Scenario 2	Scenario 3	Scenario 4
*Parameters*				
Take-up rates				
6 month- and 1 year-olds	50%	50%	67%	67%
2 year-olds	75%	75%	90%	90%
3 and 4 year-olds	90%	90%	100%	100%
Pay levels	Current	Teacher	Current	Teacher
Average pay (% of median hourly wage)	83%	134%	83%	134%
*Costing (£m)*				
Gross annual cost	26,574	40,222	32,555	49,375
(in % of GDP)	1.2%	1.9%	1.5%	2.3%

Source: author’s calculations. ‘Teacher’ pay levels signifies the pay scale of primary school teachers of equivalent qualification. ‘Current’ pay levels reflect wage rates in current commercial facilities for equivalent qualification

The gross annual investment needed appears very high. It contrasts with current levels of public subsidies for this age group at around £6 billion a year (0.28% of GDP) and to a rough estimate of £4 billion a year spent in fees directly by families (Department for Education, 2016). These figures obviously reflect both lower wages and lower use of childcare compared to the modelled provision. When calibrating the model to current wage rates, qualifications, take-up rates of formal provision and average hours, the total cost would come to about £10.3bn per year, which correspond to the private and public spending figures above. This is one way of validating the simulation parameters. The unpaid contribution by grandparents is not negligible either. The insurance company RIAS estimated that the unpaid contribution of grandparents to providing care to their grandchildren (though not just of pre-school age) amounted to about £17 billion a year in 2014, with 9.1 million grandparents providing at least one hour of childcare per week, for an average of 9.1 h per week (RIAS, 2014).

## Employment effects

### Labour demand

On the demand-side of the labour market, the main effect of the investment is to create jobs directly in the childcare sector, which will depend on the take-up scenario. This direct effect is accompanied by an indirect employment effect stemming from increased demand for inputs from other sectors (food, electricity, construction, transport, etc.), which themselves will require their own inputs from their supplying industries, and so forth. The labour intensity required to produce all these indirect inputs gives the corresponding indirect employment effect from which it is possible to derive an indirect employment multiplier (also called Type I multiplier), that is the number of direct and indirect jobs created for every direct job. A standard method of estimating such feedback effects is through input–output analysis (Antonopoulos et al., 2011; De Henau et al., 2016; Kim et al., 2019; Scottish Government, 2019).

Formally, using a symmetric input–output table with n industries (n x n matrix), the Type I employment effect ${E}_{j}$ of an increase in final demand of the output ${O}_{j}$ of industry j by £1 is calculated as follows:

$${E}_{j}=\sum_{i=1}^{n}{w}_{i}{L}_{ij},$$

where ${w}_{i}$ = the labour intensity of industry, i = total employment in industry i divided by ${O}_{i}$, ${L}_{ij}$ = the coefficient of the Leontief inverse matrix ${\left(I-A\right)}^{-1}$, where $I$ is the identity matrix and $A$ is the Direct Requirements matrix, with each element of the symmetric I–O table divided by its column total. Each element ${A}_{ij}$ represents the amount purchased by industry j from industry i, in order to produce £1 of ${O}_{j}$.

The direct employment effect is thus ${w}_{j}$, the indirect employment effect is ${E}_{j}- {w}_{j}$ and the Type I employment multiplier is $\frac{{E}_{j}}{{w}_{j}}$.

Note that employment can be measured in different ways (total headcount, full-time equivalent, employees only, etc.). In this analysis, I have used full-time equivalent (FTE) employees, because I need their wages to be able to calculate the induced employment effect.

Eurostat (2019) provides such symmetric IxI (industry by industry) tables for 2015 for each EU Member State with 64 industries. Calculating the 2015 Type I employment multiplier for the education industry in the UK yields a result of 1.21 (for FTE employees). This means that for every 1000 jobs created directly from investment in education, 210 jobs are created in the industries supplying inputs to the education sector. This model adopts the multiplier of the education industry rather than that of the social care industry (which includes childcare but also residential and domiciliary long-term care and social work services) because the profile of the new system of universal childcare with higher qualifications will tend to resemble the school system (with staff wages as the major contribution to final output).

A second type of employment effect (also known as Type II or induced employment effect) takes account of the impact of direct and indirect job creation on aggregate demand, stemming from an increase in consumption induced by the newly employed population. The method follows that of the Scottish Government (2019). It involves the same calculations as for the Type I effects, except that in this case the matrix A has been augmented by:

- a row representing the compensation of employees received in each industry j in proportion of ${O}_{j}$ and
- a column showing the share of spending of the household sector in each industry i as a proportion of the total household sector’s primary and secondary resources (derived from the national accounts).

I calculate the total increase in the compensation of employees for both the direct and indirect employment created $\left({W}_{dir}+{W}_{ind}\right)$ and inject this amount in the household sector to find the induced employment:

$${E}_{h}=\left(\sum_{i=1}^{n}{w}_{i}{L}_{ih}\right)\times \left({W}_{dir}+{W}_{ind}\right).$$

For direct employees, ${W}_{dir}$ is the total additional staff wage cost calculated in each scenario and for indirect employees, I use the average gross annual earnings (in FTE) in each industry from the ASHE 2018 data (ONS, 2019c) and augment them with the employer’s social security and pension contribution rates (13.8% and 4%, respectively).

### Labour supply

The investment in universal childcare of high quality is expected to boost mothers’ labour supply, which is expected to ‘recover’ to that of childless women with similar characteristics, as an approximation of the pattern of employment of mothers before they had their first child. Kleven et al. (2019a) showed that this was the case prior to the first child’s birth in the five European countries and the US they studied (see also Costa Dias et al., 2018 for the UK). I use the 2018–19 micro-data of the Family Resources Survey accessible from the UK Data Archive. This contains a representative cross-sectional sample of all private households in the UK and detailed information on their employment pattern, incomes and household composition. It is the survey used to feed into the UKMOD microsimulation tool hosted at Essex University (see details below in the next section).

To simulate the increase in labour supply I proceed as follows. Firstly, I assume that currently employed mothers of children aged 1–4 remain in employment and in particular, full-time working mothers all take up the new childcare offer.^6^ Secondly, I assume that part-time employed mothers take up the offer of 40 h of childcare at the average take-up rate of the scenario and those who do so change their working hours to reach full-time employment.

Thirdly I need to deal with the non-employed mothers. This is more complicated because I need to estimate a plausible change in behaviour as well as new wages while at the same time considering the remaining childcare places available and the possibility that some of the jobs created are taken up by competing candidates, chiefly childless unemployed women.

Note that for simplicity I assume that the jobs created on the demand side reflect current gender segregation in each sector so that no man takes up a ‘woman’s job’ and vice versa. This is consistent with other studies that have found that gender segregation in the industries where direct and indirect jobs were created did not change much (Kim et al., 2009; see also Ilkkaraçan & Kim, 2019).^7^ This means only childless unemployed women compete for the remaining jobs with non-employed mothers of young children. I assume that the total increase in hours of employment created by the investment, including indirect and induced jobs, determines the number of people who are able to be activated, after taking into account the increase in hours by the part-time employed mothers.

Mothers of children aged 1–4 not in employment and childless unemployed women take up the jobs in order of their employment probability ranking until the pool of jobs is exhausted, and additionally for mothers, until the number of childcare places is exhausted. This method follows that of Antonopoulos et al. (2011) and Kim et al. (2019). The ranking is estimated based on observable characteristics^8^ and a random element is added to allow for unobserved heterogeneity among otherwise similar individuals.^9^

For those moving into employment I assume that the hourly wage rate they receive is the weighted average of the wages in the newly created childcare sector in each scenario (see Table 3) and that of the wages in the industries where indirect and induced employment was created.^10^

Table 4 summarises the various employment effects for the four scenarios. The upper panel shows the number of additional jobs (in full-time equivalent) created in the childcare sector and the rest of the economy, and for each, the proportion of those assumed to go to women.^11^ The lower panel shows the impact of this job creation on men’s and women’s employment rates overall, in terms of percentage point changes, as well as on the gender gap in employment rates. The last row shows the change in the employment rate of mothers of children aged 1–4, in full-time equivalent, as a result of their increased labour supply.

**Table 4:** Employment created in the childcare industry and more widely by pay scenario

	Scenario 1	Scenario 2	Scenario 3	Scenario 4
*Parameters*				
Average take-up rate	71%	71%	85%	85%
Pay level	Current	Teacher	Current	Teacher
*Employment creation*				
ECEC services	603,000	603,000	807,000	807,000
% Women	98%	98%	98%	98%
Other sectors	258,000	340,000	341,000	441,000
% Women	48%	48%	48%	48%
Total	860,000	942,000	1,147,000	1,248,000
% Women	83%	80%	83%	80%
*% Point change in employment rates (FTE)*				
All	2.1	2.3	2.8	3.0
All men	0.7	0.9	0.9	1.2
All women	3.4	3.6	4.6	4.8
Gender gap (men–women)	− 2.7	− 2.7	− 3.6	− 3.6
Mothers of 1–4 year-olds	22.9	23.5	29.9	31.0

Source: author’s calculations; job creation figures are of employees in full-time equivalent (FTE)

The employment creation varies from 860,000 FTE jobs overall in scenario 1 to 1.2 million in scenario 4, representing a rise in employment rates of 2 percentage points and 3 percentage points, respectively. Because of the disproportionate increase in women’s employment, the gender gap in employment rates would be reduced by up to 3.6 points from a gap of 9 percentage points in 2018.^12^

The impact of the investment is mostly felt on the employment of mothers of young children, with the simulation generating an increase of 23 percentage points in scenario 1 and 31 points in scenario 4, from a baseline full-time equivalent employment rate of 46% observed in the data. Although representing a steep increase, the new employment rate of mothers would still be below that of their childless female counterparts by about ten to twelve percentage points depending on the scenario. The proportion of non-employed mothers who would move into employment varies between 32% in scenario 1 and 48% in scenario 4.

## Fiscal considerations

### Direct taxes and cash benefits

Changes to tax liabilities and cash benefits entitlements can be estimated using a microsimulation tool to account for the household and individual characteristics of the people whose employment has changed. The University of Essex hosts UKMOD, the UK part of EUROMOD, a microsimulation model that allows calculations of direct (income) taxes, social security contributions and cash benefits of households in European countries. It is free to access for everyone, provided they have access to the underlying data, which can be done via registration on the UK Data Archive (free for all researchers in recognised institutions).

The model is very flexible and allows the user to modify any aspect of the tax-benefit system that is simulated, in order to compare entitlements and liabilities. In that sense, it is a static model, assuming no behavioural response. However it is possible to modify behaviour prior to running the model by changing the employment and earnings in the input dataset, which is what I have done, as explained in the previous section.

I run each of the scenarios through the model without changing any parameter of the tax-benefit policies so that the changes in total taxes and means-tested benefits will reflect the increase in employment and earnings attributable to the childcare investment.^13^

### Non-direct taxes

The UKMOD tool does not allow simulations of indirect taxes because the Family Resources Survey does not have complete consumption data. Only childcare expenses and housing costs are recorded, for the purpose of establishing benefit entitlements. In order to add indirect taxes to the mix, I use National Statistics data on the average proportion (incidence) of indirect taxes paid out of disposable income by household income decile and apply this incidence to the average increase in disposable household income for each decile group (ONS, 2020).

In the UK, about 25% of tax revenue comes from other taxes such as capital gain taxes, property taxes and corporation taxes (IFS, 2021). These are also expected to increase due to the increase in overall economic activity (Fortin et al., 2013). However such increase is less likely to come directly from the money invested in childcare services as the model assumes public provision, hence no profits for providers and thus no corporation tax, etc. Nevertheless it is likely that the part of the economy growing out of indirect and induced activity will attract such taxes. So the remainder of the tax revenue will consist of 25% of the tax revenue borne out of the GDP increase stemming from the GDP multiplier effect. GDP increases by between 30 and 50% more than the initial investment in childcare depending on the scenario retained (whether current wages or teacher wages, respectively) and overall tax revenue is about 33% of GDP (OECD, 2020). Hence it is reasonable to assume that the remaining taxes will amount to 25% of 33% of the increase in GDP over and above the initial investment. A similar approach is used in Fortin et al. (2013).

### Total fiscal changes

Table 5 gives a summary of all the taxes calculated and changes to cash benefits as a result of the childcare investment according to each scenario, and taking account of the current ECEC subsidies (Farquharson, 2019). The last row shows the proportion of the gross annual cost that is recouped from tax revenue and reduced spending on benefits as a result of increased employment and economic activity. Despite representing significant proportions of the initial investment, the revenue recouped leaves a funding shortfall (‘net funding gap’) in all scenarios, varying between 0.3% of GDP in scenario 1 and 0.8% of GDP in scenario 4.

**Table 5:** Fiscal effects of different scenarios of universal childcare provision (2018)

	Scenario 1	Scenario 2	Scenario 3	Scenario 4
*Parameters*				
Average take-up rate	71%	71%	85%	85%
Pay level	Current	Teacher	Current	Teacher
*Costing (£ million)*				
Gross annual cost	26,574	40,222	32,555	49,375
(in % of GDP)	1.2%	1.9%	1.5%	2.3%
Direct tax revenue	7911	10,506	9819	13,916
Indirect tax revenue	2677	3518	3518	4998
Other tax revenue	651	1770	797	2172
Lower spending on cash benefits	3536	4222	4646	5899
Current ECEC subsidies	4461	4461	4461	4461
Net funding gap	7339	15,745	9314	17,929
(in % of GDP)	0.3%	0.7%	0.4%	0.8%
% self-funding	72%	61%	71%	64%

Source: own calculations using UKMOD microsimulation tool, ONS (2020) and OECD (2020). ‘Teacher’ pay levels signifies the pay scale of primary school teachers of equivalent qualification. ‘Current’ pay levels reflect wage rates in current commercial facilities for equivalent qualification. Current ECEC subsidies include free childcare entitlement for eligible 2-to-4 year-olds and tax relief on childcare expenses but excludes spending on tax credits as part of the means-tested benefits since these are simulated in UKMOD (part of ‘lower spending on cash benefits’)

### Funding the shortfall

It is not expected that the fiscal benefits of investing in high-quality universal childcare would all materialise in the short run. Most benefits are in fact expected over a long period of time in the form of increased productivity, better outcomes for children, lasting employment for mothers and consequent reduction in gender gap in earnings over the lifetime, including pension income, compared to fathers (Garcia et al., 2016). Not all of these benefits can be monetised, let alone materialise in the form of tax revenue. The main long-term benefit that can easily be simulated would be the persistent employment effect for mothers, as discussed in the overview of the modelling strategy section. This is discussed in the next section.

First, let’s examine the more usual way for government to fund any programme of investment in public services that are recurrent, which is to raise taxation contemporaneously. Given the identified short-term fiscal benefits reported in Table 5, it is possible to argue that the rise need not fund the whole investment, only the shortfall. This can be done in many ways. Arguably, due to the nature of the immediate financial benefits of high-quality childcare accruing to parents (and to workers in general given the increase in economic activity overall), it would make sense to fund a significant portion of the shortfall through increases in social security contributions paid for by earners.^14^ A smaller portion of the shortfall could be funded via general taxation, for example in the form of income tax rise given the more general benefits of childcare investment for society as a whole. This paper is not intending to fit an optimal combination of tax changes but it is possible, using UKMOD, to simulate some changes and tailor them to meet certain principles, such as keeping the average tax incidence on disposable income small, in order to avoid distortions, and to make the changes progressive overall.

I use UKMOD to simulate (by trials and errors) the tax changes necessary to make each scenario self-funding by filling the funding gap. The changes required are as follows:

- raising the first rate of National Insurance contributions (SSC) by 1 percentage point in scenarios 2 and 4 (involving teacher pay scales) and 0.5 percentage point in scenarios 1 and 3 (involving current pay scales)
- raising the second rate of SSCs by 2 percentage points in all scenarios^15^
- adjusting the first threshold of personal income tax (called the personal allowance or PA) downwards until I obtain the right level that enables the gap to be filled entirely, within a margin of 0.1%. Scenario 1 requires a reduction of the PA by 2%, Scenario 2, by 10%, Scenario 3 by 3% and Scenario 4 by 12%.^16^

Appendix Tables 7, 8, 9, 10 show the distributional results of a decomposition of the effects of the investment on childcare costs, net earnings and additional tax rises. They show that by income decile, the net effect is extremely progressive as the lowest income decile group benefits the most in proportion of their baseline disposable income after childcare costs, while richer households’ net benefits reduce gradually along the decile scale. The top 20% of households become on average net contributors in the scenarios involving teacher pay scales (scenarios 2 and 4) but by a fraction of a percentage point (at most 0.4%).

The lower panel of each table shows the decomposition by household type, confirming the redistributive aspect of the funding method from childless couples and pensioners to adults with children as well as to single working-age adults without children, as these include unemployed individuals who have been modelled to benefit from the job creation.

### Longer-term fiscal revenue

We can now turn to the longitudinal labour supply approach discussed above and see if the programme can be self-funding over time as an alternative to raising tax and social security contribution rates. As the higher employment and earnings of mothers of young children are likely to last beyond the end of the childcare years, it is possible to model the cumulative tax and benefit differential this would entail compared to the current employment outlook of all mothers. The latter is assumed to represent the lifetime employment prospects of mothers of young children in the absence of a high-quality universal childcare system. I assume that the increased earnings of mothers of young children as a result of the take-up of the universal childcare policy will remain constant in real terms over the rest of their working life, set to about 35 years, since on average mothers have their first child at about 30 and retire at 65.

Therefore, using UKMOD we can simply calculate the average annual tax and benefit differential between the two groups and calculate how many years of this revenue are needed to offset the total cost of childcare.^17^ The FRS data show that mothers have on average 1.77 children, which means that the total childcare years used on average per mother would be about 7 years.^18^ Therefore 7 years of childcare cost divided by the annual tax-benefit differential ‘repaid’ per mother gives the number of years needed for the investment to be recouped.

Table 5 shows the results for each scenario and by level of education. If left to be funded by mothers’ earnings alone in a form of personal fiscal debt to be repaid over their career, results show that it would not be possible for low- and mid-educated mothers to repay the cost as only highly educated mothers manage to offset the costs of childcare within their typical remaining working life. But the costs are entirely offset on average for all mothers, in between 21 years for scenario 3 and 31 years for scenario 2. Moreover, the higher the take-up, the more rapidly the costs can be recouped.

These results confirm the need to consider childcare as a public service that is cross-subsidised, at least between users, if not by society overall. Either way these results support a compelling argument for fiscal affordability of an ambitious programme whose funding does not require heavy contributions by any particular group as both the costs and benefits are progressively distributed (Table 6).

**Table 6:** Average number of years needed per mother to offset childcare costs

	Scenario 1	Scenario 2	Scenario 3	Scenario 4
*Parameters*				
Average take-up rate	71%	71%	85%	85%
Pay level	Current	Teacher	Current	Teacher
*Results*				
Low-educated	85.6	66.1	45.3	37.7
Mid-educated	82.0	59.5	45.5	39.0
High-educated	17.1	22.5	14.6	18.7
All mothers	26.3	30.9	20.9	23.8

Source: own calculations using FRS data and UKMOD. Low-educated mothers correspond to those having attained at most lower secondary education; mid-educated mothers are those having attained upper secondary or post-secondary education; high-educated mothers are those with a degree

## Discussion and conclusion

Despite relatively widespread agreement that current ECEC policy in the UK remains vastly inadequate to address the challenges of quality, accessibility and affordability, arguments around lack of fiscal space were often the prime reason for opposing increased public investment in the sector. The aim of this paper was to examine such claims and in doing so make a positive fiscal case. As the costings results show, investing in free universal ECEC provision of high quality requires significant amount of annual spending, about five to ten times the amount of public spending currently committed by the government.

But this spending should be seen as an investment that provides many public benefits, in both the short and longer term. High-quality formal childcare from a young age fosters children’s well-being and social and cognitive development. In the longer term, this will raise productivity in the economy through better education, social skills and greater ability to adapt to fast-changing technology-driven labour markets. It also reduces inequalities in life chances and offer opportunities to improve everyone’s quality of life as well as social cohesion. In the short term it provides employment and reduces gender inequalities in earnings. Both of these arguments justify public spending as these benefits have a public good element: building social infrastructure. Therefore even if the programme did not generate substantial fiscal revenue, general taxation should be mobilised to achieve this objective of public service and wider benefits. The fact that it does seem to ‘pay for itself’ over time or with minimum tax rises under a range of plausible scenarios might help cut through differing political views about fiscal orthodoxy.

Indeed, taking account of the tax revenue and reduced social security spending stemming from the many direct and indirect jobs created in the economy, only between £2.80 and £3.90 for every £10 spent would require additional funding on a year-on-year basis. Funding the remainder can be done with tax increases entailing minimal additional contributions for most households. Alternatively it can be done over several years without tax changes, as the results showed also that over the longer-term fiscal benefits are likely to recoup the total investment in childcare, for mothers on average, as their lifetime earnings gap is reduced.

Such reduction in gender earning gaps is also another policy objective in its own right, improving women’s economic independence and reducing gender inequalities. Maternal employment could be greatly improved if the right incentives are provided and there is a genuine system of affordable, accessible and high-quality childcare. The method developed in this paper is easily transferable to simulate ECEC reforms in other countries. As the main limitation of the research is that employment effects are simulated, there is some uncertainty around the likely take-up of childcare and ‘activation’ of mothers. Therefore further research could aim to refine the calibration of labour supply effects and resulting fiscal benefits accounting for heterogeneity of mothers in their likelihood to take up the childcare offer, for different models of increased quality. Simulating productivity gains for children over the longer term could also be done to estimate further fiscal benefits.

## Data Availability

All data used in this article are publicly available as cited in the reference list, except for the main micro-data set (the Family resources Survey) used for some of the analysis which is available to registered users of the UK Data Service (https://www.ukdataservice.ac.uk/). Input–output tables and related employment data by industry can be found on the Eurostat databank website (Eurostat, 2019) as well as ad hoc employment and earnings data from the Office for National Statistics (ONS, 2019a, 2019b and 2019c). Data on costings and staffing are found in the literature of specific studies and official documents, also available online (e.g. Department for Education). Detailed calculations were made on Excel spreadsheets and the Stata software and results are available from the author upon request. The microsimulation tool used, UKMOD, is also free/open-access for registered researchers and can be accessed via the Centre for Microsimulation and Policy Analysis at the University of Essex, UK (https://www.microsimulation.ac.uk/ukmod/access/).

## Appendix 1: Distributional results of simulated tax rises for each scenario

See Tables 7, 8, 9, and 10.

**Table 7:** Distributional results by decile group and household type for Scenario 1

	Childcare (%)	Earnings (%)	Tax (%)	Total (%)
*Income decile group*				
D1 (lowest)	0.6	21.6	− 0.2	22.0
D2	0.1	3.7	− 0.1	3.7
D3	0.3	2.7	− 0.2	2.7
D4	0.4	1.9	− 0.3	2.0
D5	0.8	1.0	− 0.4	1.4
D6	0.7	0.9	− 0.5	1.2
D7	0.8	0.7	− 0.5	1.0
D8	0.9	1.1	− 0.6	1.4
D9	0.8	0.6	− 0.8	0.7
D10 (highest)	1.1	0.7	− 1.5	0.3
*Household type*				
Single woman	0.0	1.2	− 0.6	0.7
Single man	0.0	2.1	− 0.8	1.3
Couple no child	0.0	0.4	− 1.0	− 0.6
Lone mother	3.0	2.0	− 0.3	4.6
Lone father	1.1	0.0	− 0.7	0.4
Couple with child	2.5	3.3	− 1.1	4.7
Single woman pensioner	0.0	− 0.1	− 0.1	− 0.2
Single man pensioner	0.0	0.0	− 0.2	− 0.2
Couple pensioner	0.0	0.0	− 0.2	− 0.2
All households	0.8	1.4	− 0.8	1.5

Source: own calculations using UKMOD microsimulation tool. Decile groups are established based on ranking equivalised household disposable incomes prior to the reform. ‘Childcare’ is the effect of making all childcare expenses free for those with children aged 0–4, relative to disposable income of the household after childcare costs in the baseline pre-reform scenario. ‘Earnings’ is the effect of increased employment and earnings as a result of the reform, relative to the same baseline income after childcare. ‘Tax’ is the effect of raising SSCs and lowering PA to pay for the shortfall, and ‘Total’ is the sum of the three components giving the overall net contribution (−) or benefit ( +) of the reform

**Table 8:** Distributional results by decile group and household type for Scenario 2

	Childcare (%)	Earnings (%)	Tax (%)	Total (%)
*Income decile group*				
D1 (lowest)	0.6	30.8	− 0.7	30.6
D2	0.1	5.0	− 0.4	4.7
D3	0.3	4.0	− 0.9	3.4
D4	0.4	2.3	− 1.2	1.6
D5	0.8	1.2	− 1.3	0.7
D6	0.7	1.1	− 1.5	0.4
D7	0.8	0.9	− 1.6	0.0
D8	0.9	1.3	− 1.7	0.5
D9	0.8	0.7	− 1.8	− 0.3
D10 (highest)	1.1	0.8	− 2.2	− 0.4
*Household type*				
Single woman	0.0	2.2	− 1.5	0.7
Single man	0.0	2.5	− 1.7	0.8
Couple no child	0.0	0.6	− 2.0	− 1.4
Lone mother	3.0	2.9	− 0.8	5.0
Lone father	1.1	0.0	− 1.5	− 0.4
Couple with child	2.5	4.0	− 2.1	4.4
Single woman pensioner	0.0	− 0.1	− 0.7	− 0.8
Single man pensioner	0.0	0.0	− 0.7	− 0.7
Couple pensioner	0.0	0.0	− 0.9	− 0.8
All households	0.8	1.8	− 1.7	1.0

Source: own calculations using UKMOD microsimulation tool. Decile groups are established based on ranking equivalised household disposable incomes prior to the reform. ‘Childcare’ is the effect of making all childcare expenses free for those with children aged 0–4, relative to disposable income of the household after childcare costs in the baseline pre-reform scenario. ‘Earnings’ is the effect of increased employment and earnings as a result of the reform, relative to the same baseline income after childcare. ‘Tax’ is the effect of raising SSCs and lowering PA to pay for the shortfall, and ‘Total’ is the sum of the three components giving the overall net contribution (−) or benefit ( +) of the reform

**Table 9:** Distributional results by decile group and household type for Scenario 3

	Childcare (%)	Earnings (%)	Tax (%)	Total (%)
*Income decile group*				
D1 (lowest)	0.6	29.4	− 0.4	29.6
D2	0.1	4.7	− 0.2	4.7
D3	0.3	3.7	− 0.4	3.5
D4	0.4	2.4	− 0.6	2.2
D5	0.8	1.2	− 0.7	1.4
D6	0.7	1.2	− 0.7	1.2
D7	0.8	1.0	− 0.8	1.0
D8	0.9	1.3	− 0.9	1.3
D9	0.8	0.8	− 1.0	0.6
D10 (highest)	1.1	0.8	− 1.6	0.3
*Household type*				
Single woman	0.0	1.9	− 0.8	1.1
Single man	0.0	2.5	− 1.0	1.5
Couple no child	0.0	0.6	− 1.2	− 0.6
Lone mother	3.0	3.7	− 0.5	6.1
Lone father	1.1	0.0	− 0.9	0.2
Couple with child	2.5	4.0	− 1.3	5.2
Single woman pensioner	0.0	− 0.1	− 0.3	− 0.4
Single man pensioner	0.0	0.0	− 0.3	− 0.3
Couple pensioner	0.0	0.0	− 0.4	− 0.4
All households	0.8	1.8	− 1.0	1.7

Source: own calculations using UKMOD microsimulation tool. Decile groups are established based on ranking equivalised household disposable incomes prior to the reform. ‘Childcare’ is the effect of making all childcare expenses free for those with children aged 0–4, relative to disposable income of the household after childcare costs in the baseline pre-reform scenario. ‘Earnings’ is the effect of increased employment and earnings as a result of the reform, relative to the same baseline income after childcare. ‘Tax’ is the effect of raising SSCs and lowering PA to pay for the shortfall, and ‘Total’ is the sum of the three components giving the overall net contribution (−) or benefit ( +) of the reform

**Table 10:** Distributional results by decile group and household type for Scenario 4

	Childcare (%)	Earnings (%)	Tax (%)	Total (%)
*Income decile group*				
D1 (lowest)	0.6	45.5	− 1.2	44.8
D2	0.1	7.3	− 0.6	6.9
D3	0.3	5.4	− 1.2	4.5
D4	0.4	3.1	− 1.4	2.1
D5	0.8	1.5	− 1.6	0.7
D6	0.7	1.6	− 1.8	0.6
D7	0.8	1.2	− 1.9	0.2
D8	0.9	1.7	− 2.0	0.6
D9	0.8	0.9	− 2.0	− 0.4
D10 (highest)	1.1	0.9	− 2.3	− 0.4
*Household type*				
Single woman	0.0	3.2	− 1.7	1.4
Single man	0.0	3.7	− 1.9	1.8
Couple no child	0.0	0.9	− 2.2	− 1.4
Lone mother	3.0	5.1	− 1.0	7.0
Lone father	1.1	0.0	− 1.7	− 0.5
Couple with child	2.5	5.1	− 2.3	5.2
Single woman pensioner	0.0	− 0.1	− 0.9	− 1.0
Single man pensioner	0.0	0.0	− 0.9	− 0.9
Couple pensioner	0.0	0.0	− 1.1	− 1.0
All households	0.8	2.5	− 1.9	1.4

Source: own calculations using UKMOD microsimulation tool. Decile groups are established based on ranking equivalised household disposable incomes prior to the reform. ‘Childcare’ is the effect of making all childcare expenses free for those with children aged 0–4, relative to disposable income of the household after childcare costs in the baseline pre-reform scenario. ‘Earnings’ is the effect of increased employment and earnings as a result of the reform, relative to the same baseline income after childcare. ‘Tax’ is the effect of raising SSCs and lowering PA to pay for the shortfall, and ‘Total’ is the sum of the three components giving the overall net contribution (−) or benefit ( +) of the reform

## Abbreviations

ASHE: Annual Survey of Hours and Earnings
ECEC: Early Childhood Education and Care
FRS: Family Resources Survey
FTE: Full-time equivalent
GDP: Gross domestic product
LFS: Labour Force Survey
OECD: Organisation for Economic Cooperation and Development
ONS: Office for National Statistics
SSC: Social Security Contributions
UK: United Kingdom
VAT: Value-added tax

## Footnotes

1 Note that I focus on mothers’ labour supply as the literature has repeatedly shown that fathers are little affected by changes to childcare policies (Kleven et al., 2019a, b).

2 The choice of starting the provision at six months implies a shorter maternity leave than currently available in the UK, more in line with other western European countries but still above the EU minimum of 14 weeks.

3 Note that by taking the women’s average as reference instead of the overall average, the model implies a shorter working week for all. This does not mean that the system is intended to portray childcare as a woman’s issue that needs to fit around women’s typical hours; instead it suggests a change in the working time norm for men, so as to promote more time for them to get involved in caring activities (see XXX, 2013, for a discussion). The wider implications of such a reduced working week are however beyond the scope of this paper.

4 Average hours of informal care per week were around 11, while formal care around 20 h. Both are expected to increase with better quality and free childcare provision (Department for Education, 2019).

5 This is about 17% higher than the national minimum wage, confusingly called the National Living Wage (which was £7.83 in 2018–19). The Living Wage Foundation independently calculates the Real Living Wage by estimating the minimum wage required to achieve a minimum living standard when working full-time, that is to afford a decent minimum basket of goods and services (taking account of higher living costs in London).

6 I focus on mothers of children aged 1–4 rather than 0–4 given the unlikely change in employment for mothers with a child under 1, as many will be on maternity leave for the first few months. The FRS does not allow distinguishing a child’s age in months.

7 The concentration of women as childcare workers and pre-school teachers remains very high even in countries with long-established high-quality universal provision such as Sweden (above 90%) (Eurydice, 2019).

8 Ordered probit regression of hours of work in three ordered categories (not employed, part-time, full-time) on the covariates educational dummies, age and age squared, and partner’s earnings and square of partner’s earnings (including zero if no partner). Regressions done using the software Stata MP/14.2.

9 As such it is not a labour supply model based on wage and childcare cost elasticities but a discrete reduced-form model with the main covariate being educational level, conditioned on existing employment status.

10 At the micro-level the new jobs could be taken up by the displaced workers who used to replace the non-employed mothers now assumed to remain in their pre-birth job. In practice, at the macro-level, various reshuffles will operate in the labour market resulting in the new jobs being taken up at the assumed average wages.

11 These estimates are the net (additional) employment creation in childcare and other sectors, on top of existing childcare jobs and related jobs in the wider economy. Although jobs with better pay and working conditions replace the existing childcare jobs, these would not constitute additional, new, employment.

12 This is not negligible given the gap has remained largely unchanged since 2009 (ONS, 2019a).

13 I also checked whether changing the randomisation of take-up and activation would change the results significantly. I tested by making the take-up completely randomised on the one hand, and completely parameterised on the other (that is only reflecting the estimated probabilities based on observed characteristics). In both cases, overall results do not differ qualitatively from the main model.

14 In the UK, social security contributions have increasingly become a tax on earnings, mowing away from a contribution to future social security benefits for those insured, since most of the money collected is pooled with tax revenue to spend on overall services and transfers.

15 The UK system of social security (National Insurance) contributions is regressive on its own. Employees pay 12% on their earnings above a low threshold up to an upper limit (which is about a third higher than the average full-time earnings) and 2% on the earnings above that upper limit. So the simulation increases proportionately more the lower (second) rate of 2% (to 3%) than the first rate (from 12% to either 12.5% or 13%).

16 The personal allowance (PA) stood at £11,850 in 2018–19, about a third of the average full-time earnings.

17 I also add the indirect tax using the same proportional method as for the aggregate tax revenue calculated in the previous section. However I do not add other taxes as these are difficult to allocate to individuals.

18 7.08 exactly (= 4 years per child × 1.77 children).

## References

[CR1] Allen, R., Belfield, C., Greaves E., Sharp, C., & Walker, M. (2016). The longer-term costs and benefits of different initial teacher training routes. Report R118, 15 July, London: Institute for Fiscal Studies. https://www.ifs.org.uk/publications/8368. Accessed 10 Mar 2017.

[CR2] Andresen, M. E., & Havnes, T. (2018). Child care, parental labor supply and tax revenue. *Labour Economics,**61*, 101762. 10.1016/j.labeco.2019.101762

[CR3] Antonopoulos, R., Kim, K., Masterson, T., & Zacharias, A. (2011). Investing in care: A strategy for effective and equitable job creation. Levy Economics Institute Working Paper No. 610.

[CR4] Aran, M. A., Munoz-Boudet, A. M., & Aktakke, N. (2018). Building an ex-ante simulation model for estimating the capacity impact, benefit incidence, and cost effectiveness of child care subsidies in Turkey. *International Journal of Child Care and Education Policy,**12*, 15. 10.1186/s40723-018-0052-3

[CR5] Babchishin, L., Weegar, K., & Romano, E. (2013). Early child care effects on later behavioral outcomes using a Canadian nation-wide sample. *Journal of Educational and Developmental Psychology,**3*(2), 15–29.

[CR6] Bargawi, H., Cozzi, G., & Himmelweit, S. (2017). *Lives after austerity: Gendered impacts and sustainable alternatives for Europe*. Routledge.

[CR7] Bauchmüller, R., Gørtz, M., & Würtz Rasmussen, A. (2014). Long-run benefits from universal high-quality preschooling. *Early Childhood Research Quarterly,**29*, 457–470.

[CR8] Ben-Galim, D. (2011). Making the case for universal childcare. Briefing, Institute for Public Policy Research, London.

[CR9] Brewer, M., Cattan, S., Crawford C., & Rabe, B. (2020). Does more free childcare help parents work more? IFS Working Paper W20/09. Institute for Fiscal Studies, London. https://ifs.org.uk/uploads/WP202009-Does-more-free-childcare-help-parents-work-more.pdf. Accessed 20 Oct 2021)

[CR10] Butler, A., & Rutter, J. (2016). Creating and anti-poverty childcare system. Report for the Joseph Rowntree Foundation, January.

[CR11] Cory, G. (2017). Childcare: Key policy issues. Background briefing from the UK Women’s Budget Group, March.

[CR12] Costa Dias, M., Joyce, R., & Parodiz, F. (2018). The gender pay gap in the UK: Children and experience in work. Institute for Fiscal Studies Working Paper, London, February.

[CR13] Datta-Gupta, N., & Simonsen, M. (2007). Non-cognitive child outcomes and universal high quality child care. IZA Discussion Papers, No. 3188.

[CR16] De Henau, J. (2017). Costing a feminist plan for a caring economy: The case of free universal childcare in the UK. In H. Bargawi, G. Cozzi, & S. Himmelweit (Eds.), *Lives after austerity: Gendered impacts and sustainable alternatives for Europe* (pp. 168–188). Routledge.

[CR17] De Henau, J., & Himmelweit, S. (2013). Examining public policy from a gendered intra-household perspective: Changes in family-related policies in the UK, Australia and Germany since the mid-nineties. *Oñati Socio-Legal Series,**3*(7), 1222–1248.

[CR15] De Henau, J., & Himmelweit, S. (2021). A care-led recovery from COVID-19: Investing in high-quality care to stimulate and rebalance the economy. *Feminist Economics*, 1 March 2021, pp 453–469. https://www.tandfonline.com/doi/full/10.1080/13545701.2020.1845390. Accessed 21 Oct 2021.

[CR14] De Henau, J., Himmelweit, S., Lapniewska, Z., & Perrons, D. (2016). Investing in the care economy. A gender analysis of employment stimulus in seven OECD countries. Women’s Budget Group Report to the International Trade Union Confederation, Brussels, March 2016.

[CR18] Dearing, E., Zachrisson, H. D., & Nærde, A. (2015). Age of entry into early childhood education and care as a predictor of aggression: faint and fading associations for young Norwegian children. *Psychological Science,**26*(10), 1595–1607. 26276671 10.1177/0956797615595011

[CR19] Department For Education. (2013). More great childcare. Raising quality and giving parents more choice. DfE report, January, London. https://www.gov.uk/government/uploads/system/uploads/attachment_data/file/219660/More_20Great_20Childcare_20v2.pdf. Accessed 20 Nov 2016.

[CR20] Department for Education. (2015). Review of childcare costs: the analytical report. An economic assessment of the early education and childcare market and providers’ costs. Department for Education, London, 25th November, DFE-00295-2015. https://www.gov.uk/government/publications/review-of-childcare-costs. Accessed 20 Nov 2016.

[CR21] Department for Education. (2016). Childcare and early years survey of parents 2014–15, March. https://www.gov.uk/government/statistics/childcare-and-early-years-survey-of-parents-2014-to-2015. Accessed 10 Apr 2017.

[CR22] Department for Education. (2018a). School teachers pay and conditions, September, London. https://assets.publishing.service.gov.uk/government/uploads/system/uploads/attachment_data/file/740575/School_teachers__pay_and_conditions_document_2018.pdf. Accessed 21 Oct 2019.

[CR23] Department for Education. (2018b). Childcare and early years providers survey: 2018. https://www.gov.uk/government/statistics/childcare-and-early-years-providers-survey-2018. Accessed 12 Nov 2019.

[CR24] Department for Education. (2019). Childcare and early years survey of parents: 2019. https://www.gov.uk/government/statistics/childcare-and-early-years-survey-of-parents-2019. Accessed 21 Oct 2021.

[CR25] Elson, D. (2017). A gender-equitable macroeconomic framework for Europe. In H. Bargawi, G. Cozzi, & S. Himmelweit (Eds.), *Lives after austerity: Gendered impacts and sustainable alternatives for Europe* (pp. 15–26). Routledge.

[CR26] Eurofound. (2015). *Working conditions, training of early childhood care workers and quality of services—A systematic review*. Publications Office of the European Union.

[CR27] Eurostat. (2019). Eurostat database. http://ec.europa.eu/eurostat/data/database. Accessed 10 Apr 2019.

[CR28] Eurydice, E. C. (2019). *Key data on early childhood education and care in Europe* (2019th ed.). Publications Office of the European Union.

[CR29] Farquharson, C. (2019). Early education and childcare spending. IFS Briefing Note BN258, Institute for Fiscla Studies, London. https://ifs.org.uk/uploads/BN258-Early-education-and-childcare-spending.pdf. Accessed 20 Oct 2021.

[CR31] Fortin, P. (2018). Quebec's Childcare Program at 20: How has it done, and what the rest of Canada can learn. *Inroads: Canadian Journal of Opinion* 42. https://inroadsjournal.ca/quebecs-childcare-program-20-2. Accessed 20 Oct 2021.

[CR30] Fortin, P., Godbout, L., & St-Cerny, S. (2013). L’impact des services de garde à contribution réduite du Québec sur le taux d’activité féminin, le revenu intérieur et les budgets gouvernementaux, *Revue Interventions économiques* 47 | 2013. http://journals.openedition.org/interventionseconomiques/1858. Accessed 20 Oct 2021.

[CR32] Garcia, J., Heckman J., Leaf, D., & Prados, M. (2016). The life-cycle benefits of an influential early childhood program. NBER Working Paper No. 22993, December. http://www.nber.org/papers/w22993. Accessed 10 Aug 2017.

[CR33] Harding, C., & Cottell, J. (2018). *Childcare Survey 2018*. Family and childcare trust, London. https://www.familyandchildcaretrust.org/childcare-survey-2018. Accessed 20 Feb 2019.

[CR35] Havnes, T., & Mogstad, M. (2011). No child left behind: Universal child care and children’s long-run outcomes. *American Economic Journal: Economic Policy,**3*(2), 97–129.

[CR34] Havnes, T., & Mogstad, M. (2014). Is universal child care leveling the playing field? Evidence from non-linear difference-in-differences. IZA Discussion paper 4978. http://www.econstor.eu/bitstream/10419/36832/1/62740314X.pdf. Accessed 23 Apr 2015.

[CR36] Heintz, J., Staab, S., & Turquet, L. (2021) Don't let another crisis go to waste: The COVID-19 pandemic and the imperative for a paradigm shift. *Feminist Economics*, 1 March, online, pp 470–485, 10.1080/13545701.2020.1867762?src=recsys. Accessed 21 Oct 2021.

[CR37] House of Commons. (2018). Childcare—Report of the ninth session 2017–19. House of Commons Treasury Committee, London, 28 March 2018. https://publications.parliament.uk/pa/cm201719/cmselect/cmtreasy/757/757.pdf. Accessed 19 Nov 2019.

[CR38] Huston, A. C., Bobbit, K. C., & Bentley, A. (2015). Time spent in child care: How and why does it affect social development? *Developmental Psychology,**51*(5), 621–634. 25751096 10.1037/a0038951

[CR40] Ilkkaraçan, I. (2017). A feminist alternative to austerity: The purple economy as a gender-egalitatarian strategy for employment generation. In H. Bargawi, G. Cozzi, & S. Himmelweit (Eds.), *Lives after austerity: Gendered impacts and sustainable alternatives for Europe* (pp. 27–39). Routledge.

[CR39] Ilkkaraçan, I., & Kim, K. (2019). The employment generation impact of meeting SDG targets in early childhood care, education, health and long-term care in 45 countries. ILO Working Paper, 2019, International Labour Organisation. https://www.ilo.org/gender/Informationresources/Publications/WCMS_732794/lang--en/index.htm. Accessed 13 May 2020.

[CR41] Institute for Fiscal Studies (IFS). (2021). IFS revenue composition spreadsheet, TaxLab. https://ifs.org.uk/taxlab/data-item/ifs-revenue-composition-spreadsheet

[CR42] Kim, K., Ilkkaraçan, I., & Kaya, T. (2019). Public investment in care services in Turkey: Promoting employment and gender inclusive growth. *Journal of Policy Modeling*. 10.1016/j.jpolmod.2019.05.002

[CR43] Kleven, H., Landais, C., Posch, J., Steinhauer, A., & Zweimuller, J. (2019a). Child penalties across countries: Evidence and explanations. *AEA Papers & Proceedings* 109, 122–126. https://www.henrikkleven.com/uploads/3/7/3/1/37310663/klevenetal_aea-pp_2019.pdf. Accessed 12 Nov 2019.

[CR44] Kleven, H., Landais, C., & Søgaard, J. E. (2019b). Children and gender inequality: Evidence from Denmark. *American Economic Journal: Applied Economics*. 10.1257/app.20180010

[CR45] Kornstad, T., & Thoresen, T. O. (2007). A discrete choice model for labor supply and childcare. *Journal of Population Economics,**20*, 781–803.

[CR46] Lefebvre, P., & Merrigan, P. (2008). Child-care policy and the labor supply of mothers with young children: A natural experiment from Canada. *Journal of Labor Economics,**26*, 519–548.

[CR47] Lefebvre, P., Merrigan, P., & Verstraete, M. (2009). Dynamic labour supply effects of childcare subsidies: Evidence from a Canadian natural experiment on low-fee universal child care. *Labour Economics,**16*, 490–502.

[CR48] Li, W., Farkas, G., Duncan, G. J., Burchinal, M. R., & Vandell, D. L. (2013). Timing of high-quality child care and cognitive, language, and preacademic development. *Developmental Psychology,**49*(8), 1440–1451. 10.1037/a0030613 23127299 10.1037/a0030613 PMC4034459

[CR49] Living Wage Foundation. (2019). Living wage rates. https://www.livingwage.org.uk/calculation. Accessed 12 Nov 2019.

[CR51] Lloyd, E. (2018). Underpaid and undervalued: the reality of childcare work in the UK, *The conversation*, April 20, https://theconversation.com/underpaid-and-undervalued-the-reality-of-childcare-work-in-the-uk-87413. Accessed 20 Oct 2021.

[CR50] Lloyd, E., & Potter, S. (2014). *Early childhood education and care and poverty*. London: University of East London, International Centre for the Study of the Mixed Economy of Childcare.

[CR52] Løkken, I. M., Bjørnestad, E., Broekhuizen, M. L., & Moser, T. (2018). The relationship between structural factors and interaction quality in Norwegian ECEC for toddlers. *International Journal of Child Care and Education Policy,**12*, 9. 10.1186/s40723-018-0048-z

[CR53] Mohun Himmelweit, J., Coote, A., & Hough, J. (2014). The value of childcare. New Economics Foundation, London, February.

[CR54] OECD. (2017). Starting Strong 2017. Key OECD indicators on early childhood education and care. http://www.oecd.org/edu/school/starting-strong-2017-9789264276116-en.htm. Accessed 10 Aug 2017.

[CR55] OECD. (2020). Revenue statistics—the United Kingdom. https://www.oecd.org/tax/revenue-statistics-united-kingdom.pdf. Accessed 20 Oct 2021.

[CR56] ONS. (2019a). Labour Force Survey, reference tables. Office for National Statistics. https://www.ons.gov.uk/employmentandlabourmarket/peopleinwork. Accessed 13 Nov 2019.

[CR57] ONS. (2019b). Population estimates for UK, England and Wales, Scotland and Northern Ireland, Mid-2018, reference tables. Office for National Statistics. https://www.ons.gov.uk/peoplepopulationandcommunity/populationandmigration/populationestimates/bulletins/annualmidyearpopulationestimates/mid2018. Accessed 25 Jul 2019.

[CR58] ONS. (2019c). Annual survey of hours and earnings, revised tables 2018. Office for National Statistics. https://www.ons.gov.uk/employmentandlabourmarket/peopleinwork/earningsandworkinghours. Accessed 12 Nov 2019.

[CR59] ONS. (2020). The Effects of taxes and benefits on household income, 2018–19, reference tables. Office for National Statistics. https://www.ons.gov.uk/peoplepopulationandcommunity/personalandhouseholdfinances/incomeandwealth. Accessed 27 Oct 2021.

[CR60] Penn, H. (2009). Early childhood education and Care. Key lessons from research for policy makers. Independent report submitted to the European Commission by the NESSE networks of experts. European Commission, Brussels. http://www.nesse.fr/nesse/activities/reports/ecec-report-pdf. Accessed 21 Oct 2021.

[CR61] Petitclerc, A., Côté, S., Doyle, O., et al. (2017). Who uses early childhood education and care services? Comparing socioeconomic selection across five western policy contexts. *International Journal of Child Care and Education Policy,**11*, 3. 10.1186/s40723-017-0028-8

[CR62] RIAS. (2014). Britain’s grandparent army in full force as they save Britain £17bn annually in childcare costs. Press release, 4 December 2014. http://www.rias.co.uk/about-us/news-and-press-releases/britains-grandparent-army-in-full-force-as-they-save-britain-17bn-annually-in-childcare-costs/ Accessed 27 Jun 2016.

[CR63] Scottish Government. (2019). Input-output methodology guide, Version 4, July. https://www2.gov.scot/Resource/0054/00548002.pdf. Accessed 14 Nov 2019

[CR64] Sibley, E., Dearing, E., Toppelberg, C. O., et al. (2015). Do increased availability and reduced cost of early childhood care and education narrow social inequality gaps in utilization? Evidence from Norway. *International Journal of Child Care and Education Policy,**9*, 1. 10.1007/s40723-014-0004-5

[CR65] Stephen, C., Ang, L., Brooker, L., Sylva, K., Melhuish, E., Sammons, P., Siraj-Blatchford, I., & Taggart, B. (2011). Pre-school quality and educational outcomes at age 11: Low quality has little benefit. *Journal of Early Childhood Research,**9*, 2. 10.1177/1476718X10387900

[CR66] Ünver, Ö., Bircan, T., & Nicaise, I. (2018). Perceived accessibility of childcare in Europe: A cross-country multilevel study. *International Journal of Child Care and Education Policy,**12*, 5. 10.1186/s40723-018-0044-3

[CR67] Van Lancker, W. (2013). Putting the child-centred investment strategy to the test: Evidence for the EU27. CSB Working Paper 13/01, Centre for Social Policy, University of Antwerp, January. http://www.centrumvoorsociaalbeleid.be/sites/default/files/CSB%20Working%20Paper%2013%2001_January%202013.pdf. Accessed 20 Apr 2015.

[CR68] Van Huizen, T., & Pantenga, J. (2018). Do children benefit from universal early childhood education and care? A meta-analysis of evidence from natural experiments. *Economics of Education Review,**66*, 206–222.

[CR69] Vanleenhove, P. (2013). Full childcare coverage: Higher maternal labour supply and childcare usage? EUROMOD Working Paper No. EM 19/13, November.

